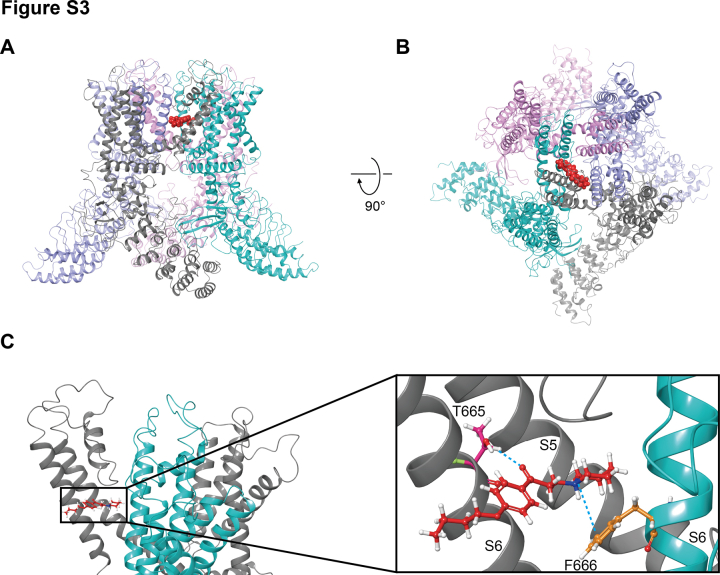# Selective inhibition of overactive warmth-sensitive Ca^2+^-permeable TRPV3 channels by antispasmodic agent flopropione for alleviation of skin inflammation

**DOI:** 10.1016/j.jbc.2023.105595

**Published:** 2023-12-26

**Authors:** Yimei Xu, Yaxuan Qu, Congxiao Zhang, Canyang Niu, Xiaowen Tang, Xiaoying Sun, KeWei Wang

**Affiliations:** 1 Department of Pharmacology School of Pharmacy, Qingdao University Medical College, Qingdao, China; 2 Medicinal Chemistry, School of Pharmacy, Qingdao University Medical College, Qingdao, China; 3 Institute of Innovative Drugs, Qingdao University, Qingdao, China

**Keywords:** TRPV3, drug repurposing, flopropione, electrophysiology, docking

## Abstract

The temperature-sensitive Ca^2+^-permeable TRPV3 ion channel is robustly expressed in the skin keratinocytes, and its gain-of-function mutations are involved in the pathology of skin lesions. Here, we report the identification of an antispasmodic agent flopropione that alleviates skin inflammation by selective inhibition of TRPV3. In whole-cell patch clamp recordings, flopropione selectively inhibits macroscopic TRPV3 currents in a concentration-dependent manner with an IC_50_ value of 17.8 ± 3.5 μM. At the single-channel level, flopropione inhibits TRPV3 channel open probability without alteration of its unitary conductance. In an *in vivo* mouse model of skin inflammation induced by the skin sensitizer DNFB, flopropione also alleviates dorsal skin lesions and ear skin swelling. Further molecular docking combined with site-directed mutagenesis reveals that two residues E501 and I505 in the channel S2-helix are critical for flopropione-mediated inhibition of TRPV3. Taken together, our findings demonstrate that the spasmolytic drug flopropione as a selective inhibitor of TRPV3 channel not only provides a valuable tool molecule for understanding of TRPV3 channel pharmacology but also holds repurposing potential for therapy of skin disorders, such as dermatitis and pruritus.

## Significance Statement

The antispasmodic agent flopropione inhibits cutaneous overactive TRPV3 channels and may hold repurposing potential for the prevention or therapy of TRPV3-related skin diseases such as dermatitis and chronic pruritus. Flopropione also can serve as an essential pharmacological tool for further understanding TRPV3 pharmacology.

As a member of the thermoTRP channel subfamily, the transient receptor potential vanilloid-3 (TRPV3) is a warmth-sensitive Ca^2+^-permeable non-selective cation channel that is primarily expressed in the skin keratinocytes (1, 2, 3, 4, 5). The recent cryo-EM structures of TRPV3 have confirmed the formation of a homologous tetramer of the channel (6, 7, 8), with symmetrical arrangement of four subunits around the central pore and each subunit consisting of six transmembrane α-helical domains (S1∼S6), an intracellular N terminal domain (NTD), and a C terminal domain (CTD) (9, 10). It has been shown that this temperature-sensitive and ligand-gated polymodal TRPV3 channel plays a critical role in skin physiology, such as skin barrier, skin sensation, and wound healing (11, 12, 13, 14). Moreover, the overactive TRPV3 function caused by either genetic gain-of-function mutations or skin sensitizers is implicated in the pathology of skin disorders such as hyperkeratosis, dermatitis, chronic pruritus, and abnormal hair growth (1, 15). These observations highlight the importance of understanding TRPV3 channel pharmacology and identifying specific inhibitors, which may hold potential for therapy of skin diseases.

Several plant-derived compounds including osthole and forsythoside B have been identified as inhibitors of the TRPV3 channel and they have been used as treatments for inflammatory diseases (16, 17). The recent identification of the TRPV3 inhibitor dyclonine, an active ingredient of throat lozenge, not only provides another useful tool molecule for research but also suggests its potential for repurposing as a therapeutic agent (18, 19). Drug repurposing is an increasingly adopted and efficient strategy for identifying approved and investigational drugs for new therapies, benefiting from their known safety profiles, reduced development times, and cost efficiencies (20, 21, 22, 23).

In this study, we investigated the effect of an antispasmodic agent flopropione, a derivative of acyl phloroglucinol isolated from *Inula viscosa* (24), on TRPV3 channels. *Inula* *viscosa* (L.) Ait. (Tribus Inulea, Compositeae) is a perennial medicinal plant widely used in traditional medicine to treat a variety of ailments including skin diseases, wounds, hypertension, diabetes, cancer, bronchitis, and gastroduodenal diseases (25, 26, 27, 28). Flopropione is also previously known to act by inhibiting catechol-O-methyltransferase (COMT) and antagonizing serotonin receptors (29). In this study, we report a novel role for flopropione in specific inhibition of TRPV3 channel, and we demonstrate that topical application of flopropione alleviates skin lesions.

## Results

### Identification of antispasmodic flopropione as a selective TRPV3 inhibitor in patch-clamp recordings

**Figure 1:**
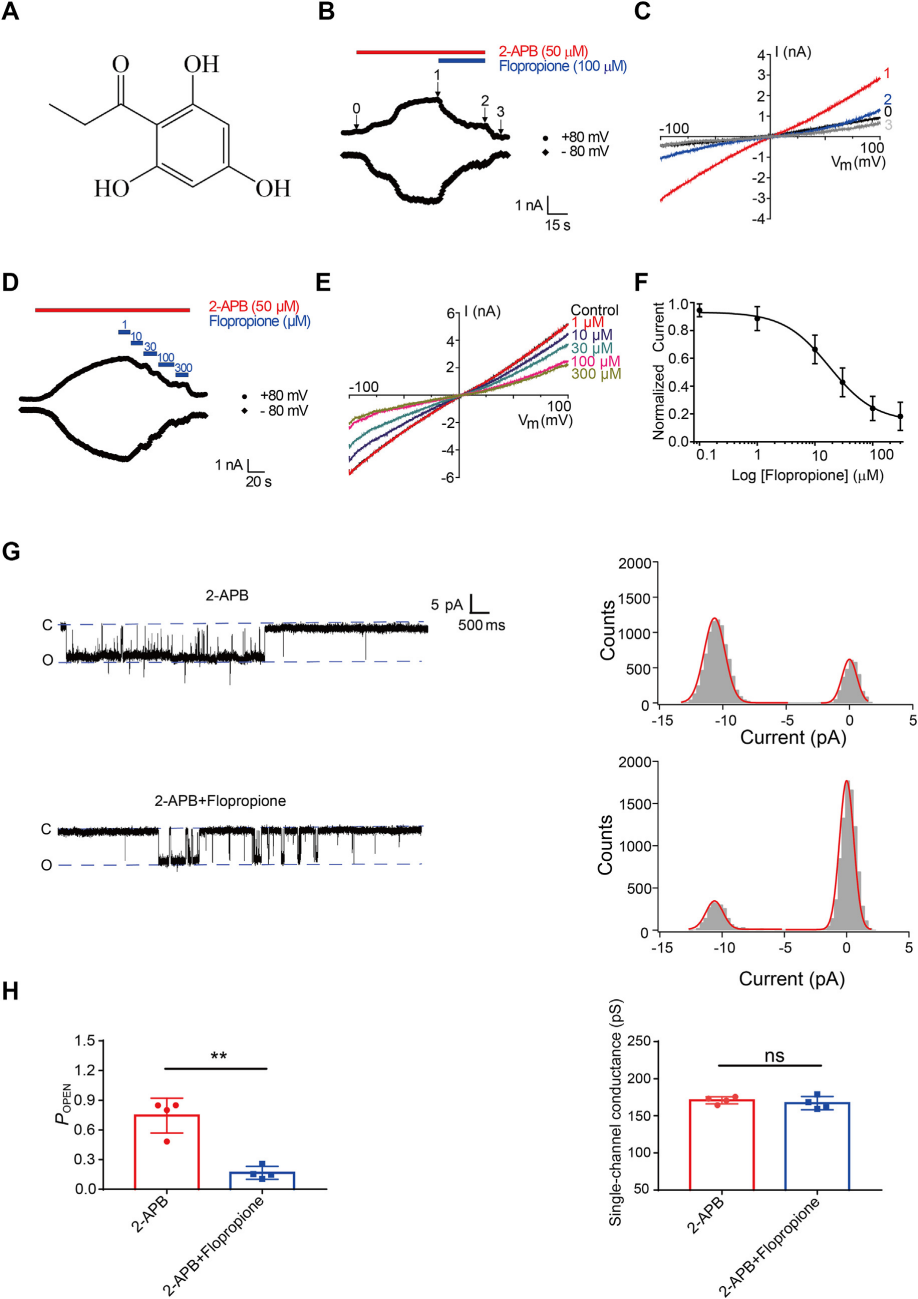
**Concentration-dependent inhibition of TRPV3 currents by flopropione**. *A*, chemical structure of flopropione. *B*, whole-cell patch clamp recordings of TRPV3 channel currents expressed in HEK293 cells in response to 2-APB alone (50 μM, *red bar*) or co-application of 100 μM flopropione (*blue bar*). *C*, current-voltage curves of TRPV3 in response to voltage ramps from −100 to +100 mV before (0) and after 50 μM 2-APB (1) and co-application of 50 μM 2-APB and 100 μM flopropione (2) and washout (3). *D*, inhibition of whole-cell currents by increasing concentrations of flopropione at 1 to 300 μmol. *E*, current-voltage curves of TRPV3 in response to voltage ramps from −100 to +100 mV after 50 μM 2-APB and different concentrations of flopropione. *F*, Hill equation fitting of dose-dependent inhibition of 2-APB-mediated TRPV3 activation by flopropione with an IC_50_ of 17.8 ± 3.5 μM (n = 5). Data are presented as the mean ± SD. *G*, *left panels*, representative single-channel current traces recorded at −60 mV in inside-out configurations before and after the addition of 30 μM 2-APB or co-application with 50 μM flopropione through bath. All-point amplitude histograms of single-channel currents in 1000-ms were shown in their *right panels*. *Dotted lines* indicate the opened channel state (O) and the closed channel state (C), respectively. *H*, summary for hTRPV3 single channel open probability (*P*_OPEN_) values and single channel conductance in the presence of different TRPV3 modulators (n = 4, ∗∗*p*< 0.01, paired *t* test). Data are shown as the mean ± SD; ns, no significance.

We started virtual screening of a library that contains 813 FDA-approved drugs against the cryo-EM structure of TRPV3 using Schrödinger. Among the identified compounds, we identified 50 drugs with docking scores above −4, as compared with known TRPV3 inhibitors used as controls. To assess the effects of these drugs on hTRPV3 channels, we carried out whole-cell patch-clamp recordings of the HEK293 cells expressing hTRPV3 channels. Perfusion of flopropione at a concentration of 100 μmol/L resulted in an approxiamtely 84% inhibition of TRPV3 currents activated by agonist 2-APB at 50 μmol/L before washout (Fig. 1, *A*–*C*). Additionally, we determined the dose-dependent inhibition of TRPV3 currents by flopropione with an IC_50_ value of 17.8 ± 3.5 μM (Fig. 1, *D*–*F*).

To further confirm the direct effect of flopropione on single TRPV3 channels, we performed the single-channel recordings of HEK293 cells overexpressing TRPV3 channels in the inside-out configuration. As a control, perfusion of agonist 2-APB at 30 μmol/L activated the single TRPV3 channel currents with single-channel conductance of about 170.9 ± 4.8 pS and open probability of 0.75 at a holding potential of −60 mV (Fig. 1*G*). In contrast, when flopropione was perfused in the presence of 2-APB at 30 μM, it resulted in the reduction of single-channel open probability to 0.17 ± 0.06 from 0.75 without any significant alteration of unitary single channel conductance of 167.1 ± 9.0 pS before washout (Fig. 1*H*). These results demonstrate that flopropione acts directly on individual TRPV3 channels by reducing the channel open probability.

**Figure 2:**
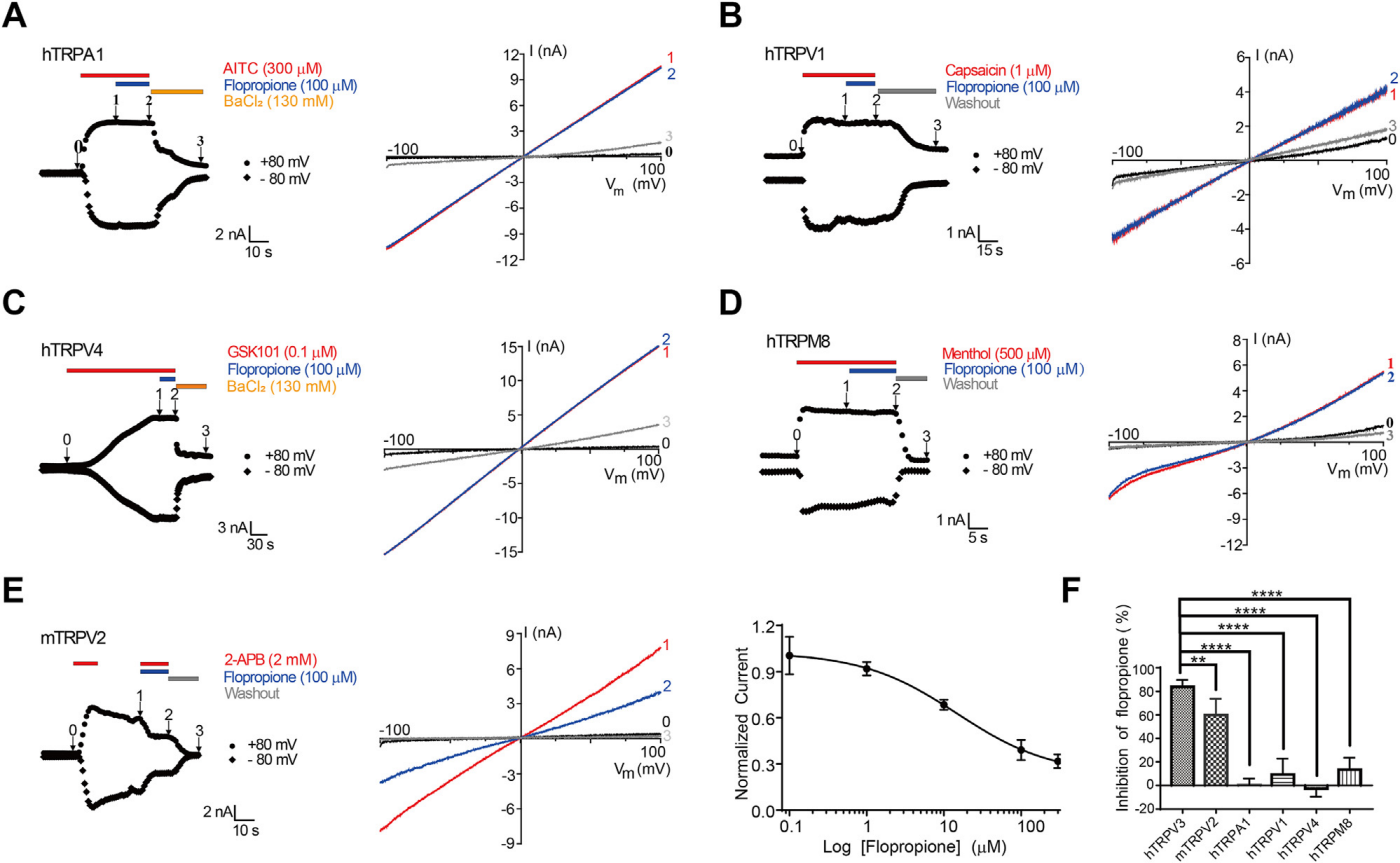
**Lack of inhibition of TRPA1, TRPV1, TRPV4 and TRPM8 channel currents by flopropione**. *A*, whole-cell TRPA1 currents were activated by 300 μM AITC (*red bar*) and co-application of 100 μM flopropione (*blue bar*) and inhibited by 130 mM BaCl_2_. *Right panel*: Current–voltage (I-V) curves of TRPA1 in response to voltage ramps from −100 to +100 mV before (0) and after 300 μM AITC (1) and co-application with 100 μM flopropione (2) and inhibited by BaCl_2_ (3). *B*, TRPV1 currents were evoked by 1 μM capsaicin (*red bar*) and its co-application with 100 μM flopropione (*blue bar*) and washout. *Right panel*: I-V curves of TRPV1 in response to voltage ramps from −100 to +100 mV before (0) and after 1 μM capsaicin (1) and co-application of 1 μM capsaicin and 100 μM flopropione (2) and washout (3). *C*, TRPV4 currents were activated by 0.1 μM GSK1016790A (GSK101; *red bar*), and co-application of 100 μM flopropione (*blue bar*) and inhibited by 130 mM BaCl_2_. *Right panel*: Current–voltage curves of TRPV4 in response to voltage ramps from −100 to +100 mV before (0) and after 0.1 μM GSK101 (1) and co-application with 100 μM flopropione (2) and inhibited by BaCl_2_ (3). *D*, TRPM8 currents were elicited by 500 μM menthol (*red bar*) and in the presence of 100 μM flopropione (*blue bar*) and washout. *Right panel*: I-V curves of TRPM8 in response to voltage ramps from −100 to +100 mV before (0) and after 500 μM menthol (1) and co-application with100 μM flopropione (2) and washout (3). *E*, *left panel*: Moderate inhibition of 2 mM 2-APB-activated TRPV2 currents (*red bar*) by co-application of 100 μM flopropione (*blue bar*) and washout. *Middle panel*: I-V curves of TRPV2 in response to voltage ramps from −100 to +100 mV before (0) and after 2 mM 2-APB (1) and co-application of 2 mM 2-APB and 100 μM flopropione (2) and washout (3). *Right panel*: Hill equation fitting of dose-dependent inhibition of 2-APB-mediated TRPV2 activation by flopropione with an IC_50_ of 14.8 ± 6.6 μM (n = 5). Data are presented as the mean ± SD. *F*, summary of inhibition of hTRPV3, hTRPA1, hTRPV1, mTRPV2, hTRPV4 and hTRPM8 currents by 100 μM flopropione. Data are shown as the mean ± SD.; n = 3 to 6; ∗∗*p* < 0.01, ∗∗∗∗*p* < 0.0001, by one-way ANOVA, followed by the Dunnet’s test.

To determine the selective inhibition of TRPV3 currents by flopropione, we conducted further tests on other subtypes of thermoTRP channels such as TRPV1, TRPV2, TRPV4, TRPM8, and TRPA1 channels transiently expressed in HEK293 cells. As shown in Figure 2*B*, flopropione at 100 μM had no effect on TRPV1 as compared with the positive control of 1 μM agonist capsaicin activating TRPV1. We also examined the effect of flopropione at 100 μM on TRPV4, TRPM8 and TRPA1 currents that elicited by their agonists GSK1016970 A (0.1 μM), menthol (500 μM), and AITC (300 μM), respectively, and flopropione had no effect on those channels (Fig. 2, *A*, *C*, and *D*). However, when testing the effect of flopropione (100 μM) on TRPV2, we observed approximately 60% inhibition of TRPV2 currents activated by 2-APB at 2 mM (Fig. 2*E*). In addition, we determined the dose-dependent inhibition of TRPV2 currents by flopropione with an IC_50_ value of 14.8 ± 6.6 μM (Fig. 2*E*).

**Figure S1:**
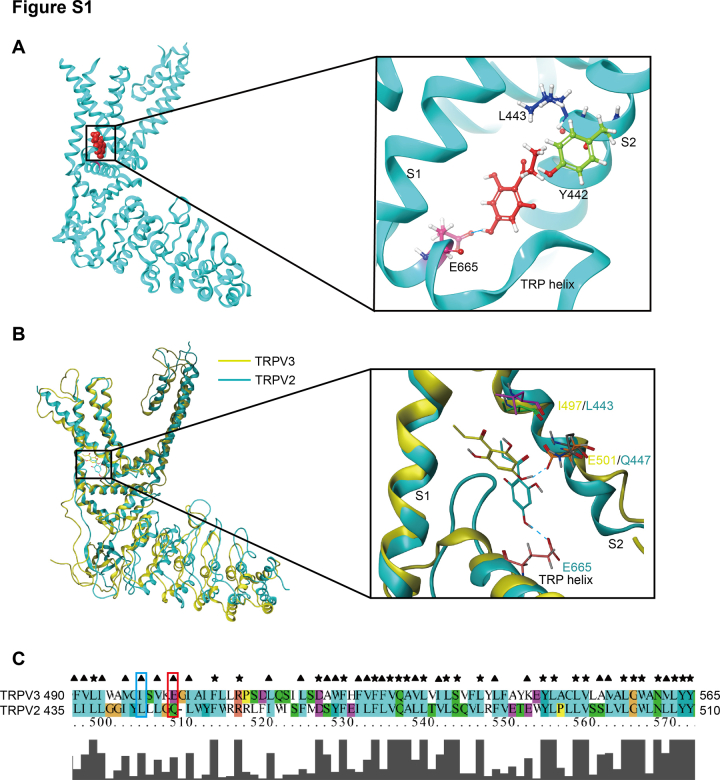


Further docking of flopropione into TRPV2 protein (PDB ID code: 7XEV) revealed a putative flopropione binding pocket formed by residues Y442, L443 from S2 and E665 from TRP helix of one subunit (Fig. S1*A*). These results indicate that flopropione is a relatively selective TRPV3 inhibitor over other subtypes of TRPV1, TRPV4, TRPM8, and TRPA1 channels, with the exception of moderate inhibition of TRPV2 by flopropione.

### Identification of TRPV3 residues critical for flopropione binding

**Figure 3:**
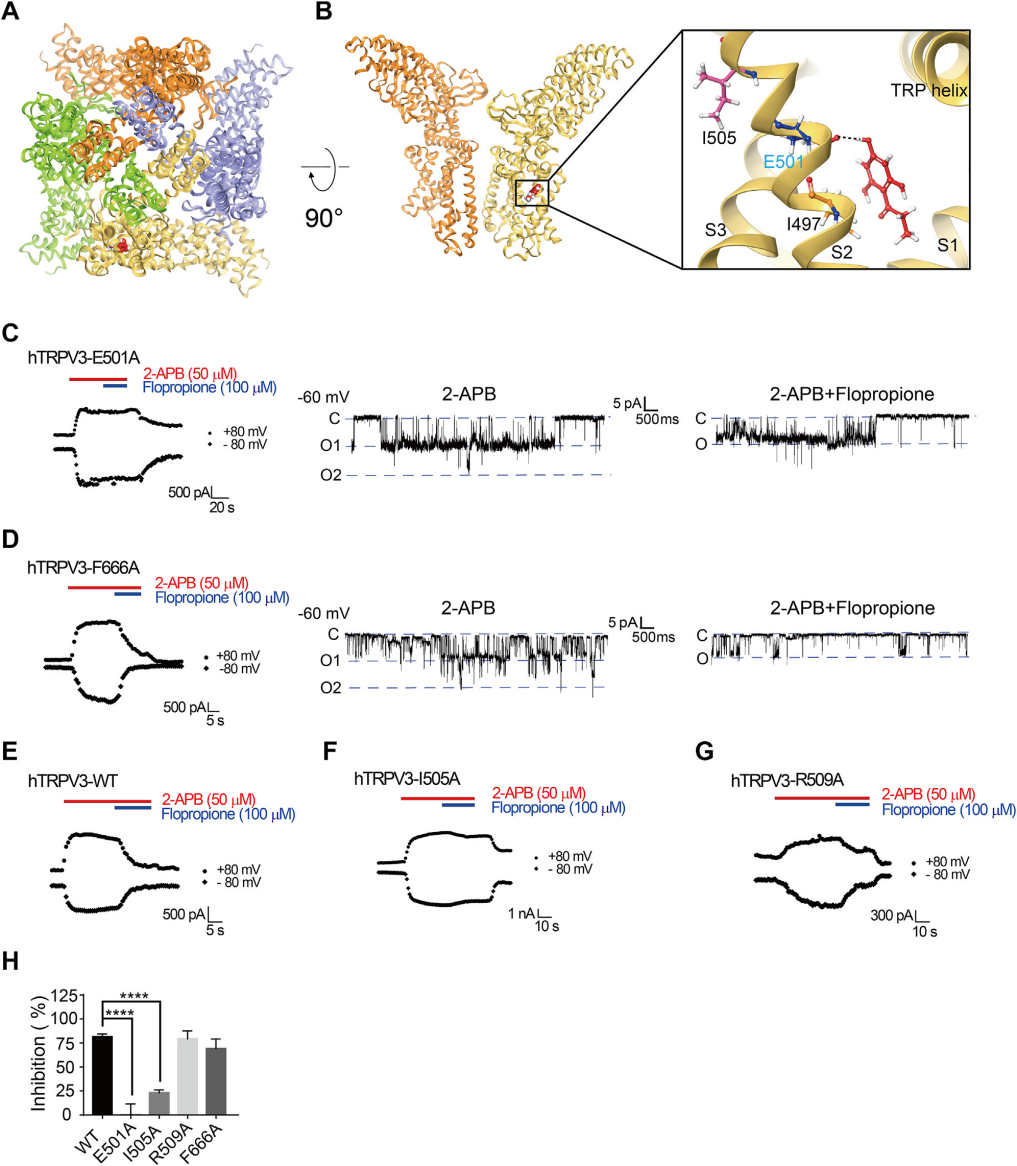
**Identification of residues critical for flopropione binding to and inhibiting TRPV3 channels**. *A*, a panoramic view of a putative pocket for flopropione binding to TRPV3 structure (PDB ID code: 6DVY) from docking. The four subunits of the tetramer are distinguished in four different colors, with bound flopropione shown in *red*. *B*, *left panel*, the side view showing only two subunits and a putative pocket for flopropione binding to the pocket; *Right panel*, an extended view of flopropione (*red*) with single TRPV3 subunit in the pocket formed by three residues I497, E501, and I505 from the S2 with the key residue E501 shown in *cyan* and a hydrogen bond is shown in *black dotted line*. *C*, *left panel*, whole-cell recordings of TRPV3-E501A mutant in response to 50 μM 2-APB alone or 2-APB with 100 μM flopropione; Representative single-channel current traces recorded at −60 mV after the addition of 30 μM 2-APB alone (*middle panel*) or 2-APB with 50 μM flopropione (*right panel*). *D*, *left panel*, whole-cell recordings of TRPV3-F666A mutant in response to 50 μM 2-APB alone and 2-APB with 100 μM flopropione; Representative single-channel current traces recorded at −60 mV after the addition of 30 μM 2-APB alone (*middle panel*) or 2-APB with 50 μM flopropione (*right panel*). *E–G*, representative whole-cell currents of wild-type hTRPV3 (*E*), I505A (*F*), and R509A (*G*) mutants in responses to 2-APB alone and 2-APB with flopropione. *H*, summary for WT hTRPV3 or mutant channel current inhibition by 100 μM flopropione (n = 3–6). Data are shown as the means ± SD.; ∗∗∗∗*p* < 0.0001, by one-way ANOVA, followed by the Dunnet’s test.

**Figure S2:**
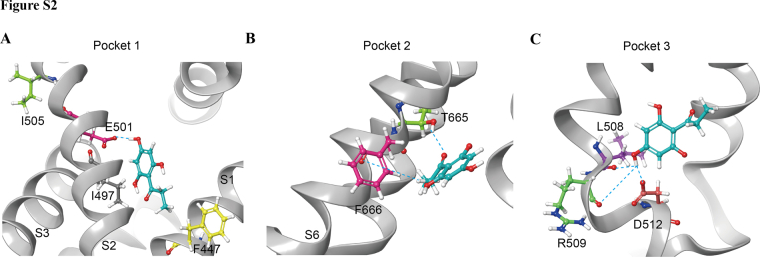


To identify the residues critical for TRPV3 inhibition by flopropione, we utilized the cryo-EM structure of mouse TRPV3 bound to 2-APB (PDB: 6DVY) and performed the docking of flopropione into the structure using Schrödinger (9). The docking results revealed three binding pockets for flopropione with the highest docking score at −5.5 kcal/mol (Fig. S2). Further analysis of binding pose and energy decomposition indicates that flopropione binds to the S2 helix through an evident hydrogen bond, with the residue E501 contributing a binding energy of −5.7 kcal/mol, which is the largest contribution compared to other amino acid residues involved (Fig. 3, *A* and *B*). In addition, the residues I497 and I505 also can assist the ligand binding. As a control, we also performed the docking with dyclonine and obtained a score of −4.5 kcal/mol (Fig. S3).

To further confirm whether the residue E501 is critical for flopropione binding, we generated site-directed mutations in the binding pocket and tested the effects of flopropione on those channel mutants. As shown in Figure 3*C*, whole-cell or single-channel recordings showed that mutating residue E501 (E501A) significantly reduced TRPV3 inhibition by flopropione. Similarly, mutating residue I505 also partially reduced TRPV3 inhibition by flopropione (Fig. 3*F*). In contrast, mutating F666 (F666A) had no effect on flopropione-mediated TRPV3 inhibition (Fig. 3*D*). Additionally, we introduced a mutation R509A in the pocket 3, which still maintained the sensitivity to flopropione inhibition, similar to the wild-type channel (Fig. 3*G*). These results demonstrate that the residues E501 and I505 in the S2 of TRPV3 channel are critical for interacting with flopropione (Fig. 3*H*).

### Topical application of flopropione alleviates skin inflammation induced by skin sensitizer activating cutaneous TRPV3

**Figure 4:**
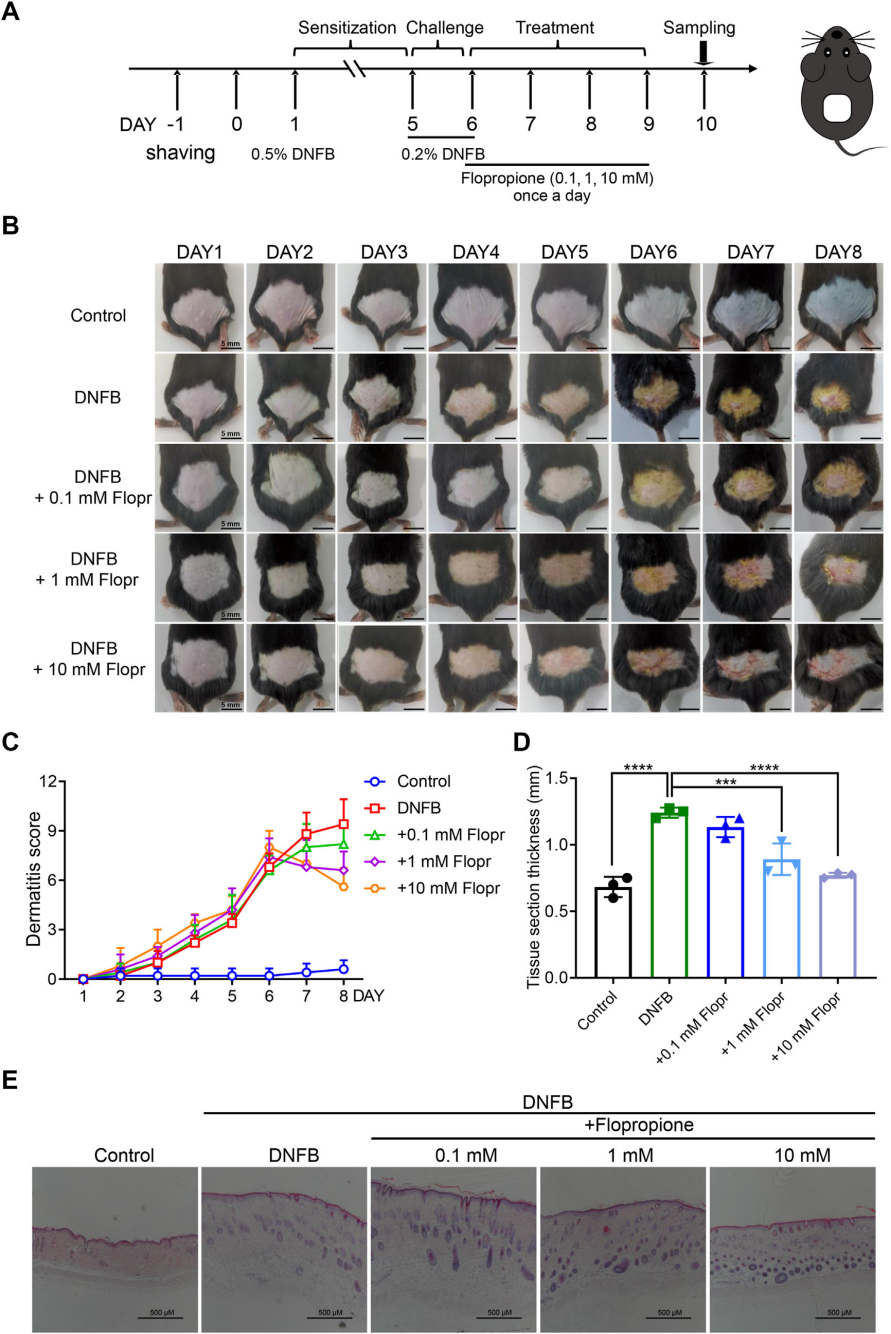
**Inhibition of skin sensitizer DNFB-induced atopic dermatitis by flopropione**. *A*, a flowchart for generation of a mouse model of dorsal skin atopic dermatitis (AD) induced by topical applications of DNFB before and after topical flopropione at different concentrations. Topical 0.5% DNFB in 100 μl was applied to dorsal skin once for skin sensitization 1 day after acclimatization and without any treatment for next 3 days. On the day five and six, topical 0.2% DNFB in 50 μl was applied to the same site once a day for 2 days for challenge. Different concentrations of flopropione in 100 μl were topically applied once a day for four consecutive days. *B*, phenotypic observation of mouse dorsal skin for eight consecutive days in the groups of solvent control, 0.2% DNFB alone, and 0.2% DNFB with flopropione (flopr) at 0.1 mM, 1 mM and 10 mM. *C*, dermatitis scores of mice in different groups treated with or without different concentrations of flopropione for eight consecutive days from *Panel B* (n = 5). ∗∗∗∗*p* < 0.0001, by two-way ANOVA. *D*, statistical analysis of the thickness of dorsal skin sections of different groups of mice (n = 3–4). ∗∗∗*p* < 0.001, ∗∗∗∗*p* < 0.0001, by one-way ANOVA, followed by the Dunnet’s test. All data are expressed as the means ± SD. *E*, representative histological H&E staining images of paraffin-embedded sections (6 μm) of mouse dorsal skin before and after DNFB or in the presence of different concentrations of flopropione. Scale bar = 500 μm.

It has been shown that the suppression of overactive TRPV3 channel function through gene silencing or the use of channel inhibitors reduces skin lesions (30, 31). We, therefore, generated a mouse model of dorsal skin inflammation induced by topical applications of skin sensitizer DNFB at a concentration of 0.5% once a day on the first day and subsequent 0.2% DNFB once a day for 2 days (Fig. 4*A*) (32). As shown in Figure 4*B*, topical applications of 0.2% DNFB resulted in a time-dependent development of dorsal skin inflammation as compared with the vehicle control (acetone/olive oil = 4/1, v/v). In contrast, topical applications of flopropione at different concentrations (0.1–10 mM) alleviated the skin inflammation in concentration-dependent manner (Fig. 4*B*) and significantly reduced the dermatitis scores, as compared with the DNFB alone group (Fig. 4*C*).

We further carried out histological examinations of dorsal skin tissue sections and found that topical flopropione at different concentrations (0.1 ∼ 10 mM) reduced mouse skin hyperkeratosis and lesion induced by DNFB, as compared with the DNFB alone group or the vehicle control (Fig. 4, *D* and *E*), which are consistent with above phenotypic observations.

**Figure 5:**
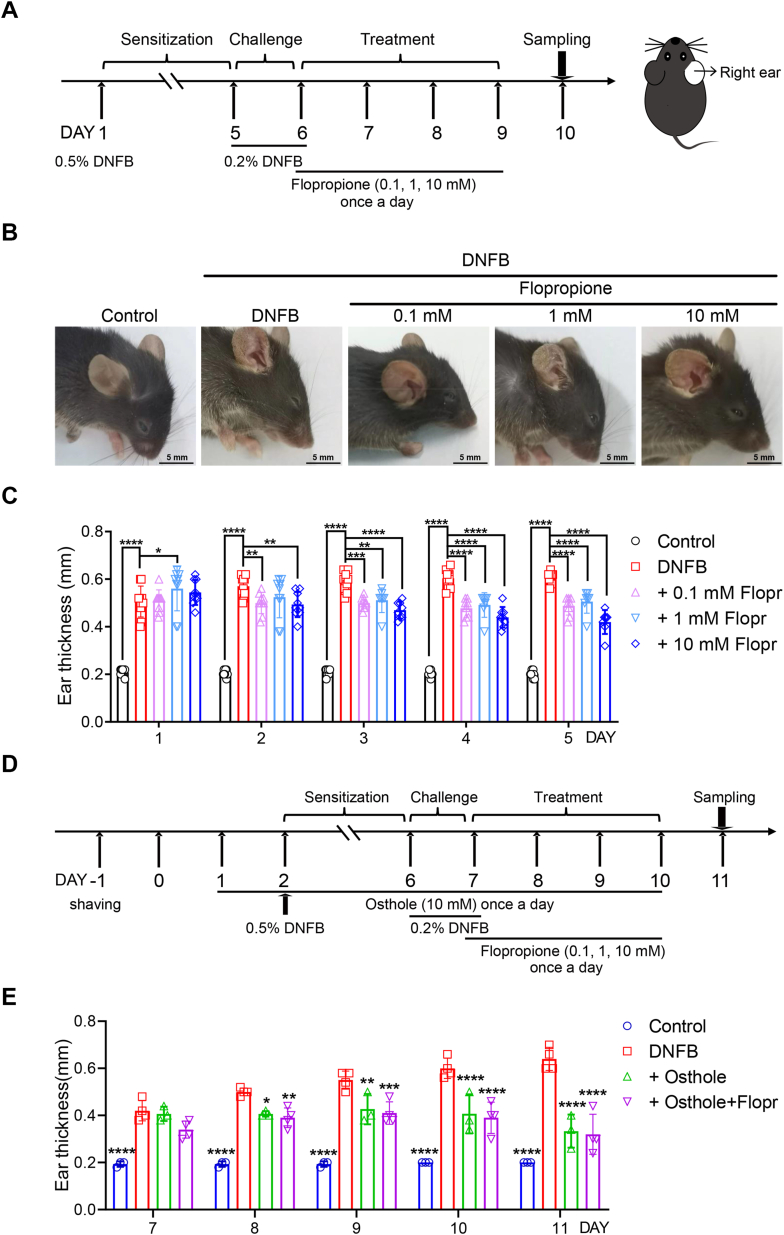
**Attenuation of DNFB-induced ear swelling by topical flopropione**. *A*, a flowchart of DNFB-induced ear swelling in mice treated with topical flopropione at different concentrations for four consecutive days. Topical 0.5% DNFB in 100 μl was applied to the right ear once for ear swelling without any treatment for next 3 days. On the fifth and sixth days, topical 0.2% DNFB in 50 μl was applied to the ear for challenge once a day for 2 days. Different concentrations of flopropione in 100 μl were topically applied once a day for four consecutive days. *B*, representative images for mouse ear swelling at day 9 before and after topical 0.2% DNFB and topical treatment of flopropione at 0.1 mM, 1 mM and 10 mM. *C*, summary of ear thickness for DNFB-induced ear swelling in mice treated with flopropione at different concentrations. Mouse ear thickness was measured daily for continuous 5 days from day 6 to 10 using Vernier caliper. Statistical significance was analyzed using by two-way ANOVA (n = 6–10). Asterisks represent the difference between Control, 0.1 mM flopropione，1 mM flopropione，10 mM flopropione and DNFB group (∗*p* < 0.05, ∗∗*p* < 0.001, ∗∗∗*p* < 0.001, ∗∗∗∗*p* < 0.0001). Data are presented as the means ± SD. *D*, a flowchart for generation of mouse model of ear swelling induced by DNFB and topical application of osthole 1 day prior to 0.5% DNFB and flopropione. *E*, summary of ear thickness for DNFB-induced ear swelling in mice. Mouse ear thickness was measured daily for continuous 11 days using Vernier caliper. Statistical significance was analyzed using by two-way ANOVA (n = 3–4). Asterisks represent the difference between Control, 10 mM osthole, 10 mM flopropione and DNFB group (∗*p* < 0.05, ∗∗*p* < 0.001, ∗∗∗*p* < 0.001, ∗∗∗∗*p* < 0.0001). Data are presented as the means ± SD.

We also assessed the effect of flopropione on ear swelling. Topical applications of 0.5% DNFB once a day and subsequent 0.2% DNFB induced ear skin inflammation (Fig. 5, *A* and *B*). In contrast, topical applications of flopropione at different concentrations on right ear for 4 days significantly reduced ear swelling, as measured daily using a vernier caliper, compared with the groups of DNFB alone or the vehicle control (Fig. 5, *B* and *C*). We further used another TRPV3 channel pore blocker osthole and tested its effect on development of DNFB-induced skin inflammation. The results showed similar attenuation of skin inflammation by either osthole alone or its co-application with flopropione, further confirming the flopropione-mediated alleviation of skin inflammation through inhibition of TRPV3 channels (Fig. 5, *D* and *E*). All these results demonstrate that inhibition of TRPV3 by flopropione alleviates skin inflammation.

## Discussion

In this study, we started the virtual docking of approved drugs into the recent cryo-EM structure of the mTRPV3 channel (PDB: 6DVY) and identified an antispasmodic agent called flopropione. The antispasmodic flopropione exhibits relative selectivity in inhibiting both macroscopic and single-channel TRPV3 currents, although it shows moderate inhibition of TRPV2. To understand the basis of this cross-inhibition of both TRPV3 and TRPV2 by flopropione, we perform the docking of flopropione into TRPV2 with binding affinity at −4.8 kcal/mol, which is lower than that of −5.5 kcal/mol with TRPV3. Moreover, we conduct the comparison of flopropione-binding pockets and their amino acids between mTRPV3 and mTRPV2 (PDB: 7XEV) (Fig. S1, *B* and *C*). We find that the residue I497 in TRPV3 corresponding to TRPV2 L443 contributes to hydrophobic interactions and the binding of flopropione, with interaction energies of −3.2 kcal/mol and −1.1 kcal/mol, respectively. Furthermore, the key residue E501 in the S2 of TRPV3, forming a hydrogen bond with the phenol hydroxyl group of flopropione with the interaction energy of −5.7 kcal/mol, plays a critical role in the flopropione-medicated inhibition of TRPV3. The residue E665 in TRPV2 can also form such a key hydrogen bond with flopropione, but it only contributes −4.4 kcal/mol affinity, which is less than that of E501 in TRPV3. On the other hand, the Q447 residue in TRPV2, corresponding to the E501 in TRPV3, is unable to form such a hydrogen bond with flopropione with negligible contribution of only −0.4 kcal/mol. As a result, flopropione exerts a relative weak inhibition on TRPV2.

As biological sensors, the polymodal biological TRP channels are not only modulated by environmental temperature but also extremely sensitive to a wide variety of chemical stimuli, including natural compounds and synthetic small molecules. It is known that the voltage sensing domains (VSD) formed by the S1-S4 transmembrane α-helices can have an impact on the pharmacology of TRP channels (7). The structural adaptability of VSD of different TRP channels may serve as a potential pharmacological target for both structure-based compound screening and disease-related drug design (7, 33, 34). Currently, there are three reported sites/domains in TRPV3 that are important in mediating the channel inhibitor binding. The first binding site is the residue Y564 located at the S4-helix, which is critical for osthole (35) and citrusinine-II inhibiting TRPV3 (36). Interestingly, this residue is also the same binding site for agonist 2-APB (35). The second binding one is the residue F666 located at the S6-helix important for the channel opening and interaction with inhibitors such as dyclonine, isochlorogenic acid A and B (37), and scutellarein (30). The third site for trpvicin binding and stabilizing the channel in closed state is involved in two residues of A556 at the S4 and A560 at the S5 important for inhibitor trpvicin binding and stabilizing the channel in the closed state (38). In this study, our findings show that flopropione binds to the pocket primarily formed by residues E501 and I505 near the S2 and its phenolic hydroxyl group of flopropione serves as a key pillar for hydrogen bonding with E501 residue.

Overactive TRPV3 caused by its gain-of-mutations or skin sensitizers is implicated in skin diseases. Therefore, targeting TRPV3 with approved or investigational drugs has repurposing potential. It is worth noting that the current existing TRPV3 inhibitors are either lack of selectivity or insufficiently evaluated against other members of TRP subfamily. These include natural products such as forsythoside B (17), osthole (16), verbascoside (39), isochlorogenic acid A and B (37), citrusinine-II (36), scutellarein (30) and small molecules such as 17(R)-resolvin D1 (40), 26E01 (41) and dyclonine (18). The inhibitor osthole inhibits TRPV3 with an IC_50_ value of approximately 37.0 μM, and it also exhibits weak inhibition of TRPV1 and TRPV4 (16, 42). Natural phenylethanoid glycoside forsythoside B inhibits TRPV3 currents with an IC_50_ of about 6.7 μM and also exhibits weak inhibition of TRPA1 currents (17). The anesthetic dyclonine inhibits TRPV3 currents with IC_50_ about 3.2 μM and also inhibits TRPV2 currents (IC_50_ of 31 μM) and TRPM8 currents (IC_50_ of 82 μM) (18).

In conclusion, we demonstrate that the antispasmodic agent flopropione inhibits TRPV3 channels and alleviates skin inflammation and injury. Flopropione not only provides a molecular tool but also holds repurposing potential for the prevention or therapy of TRPV3-related skin diseases such as dermatitis and chronic pruritus.

## Experimental procedures

### Chemicals

Compounds flopropione (MW: 182.18) and osthole (MW: 244.29) were purchased from TargetMol. Compounds 2-aminoethoxydiphenyl borate (2-APB), carvacrol (Car), capsaicin, menthol, GSK1016790 A (GSK101), and allyl isothiocyanate (AITC) were purchased from Sigma-Aldrich. Compounds were made in DMSO as stock solutions before use. Compounds used for patch-clamp recordings were diluted in perfusion solution. Compounds used for generation of atopic dermatitis and ear swelling models were diluted in the solvent of acetone/olive oil = 4/1, v/v before topical applications.

### Animals

C57BL/6J mice (male, 6–8 weeks old, 20 ± 2 g) were purchased from Beijing Vital River Laboratory. All mice were acclimated for at least 1 week before experiments for their adaptation to new environment where the temperature was maintained at 22 ± 2 °C with a normal 12-h circadian cycle and free access of food and water. All animal tests were approved by the Institutional Animal Care and Use Committee of Qingdao University Health Science Center.

### Cell transfection and culture

HEK293 cells were cultured in Dulbecco’s modified Eagle’s medium (DMEM, Gibco) containing 4.5 g/L D-glucose supplemented with 10% fetal bovine serum based at 37 °C with 5% CO_2_. For whole-cell patch-clamp recordings, cells were grown in glass coverslips for 24 h before transient transfections with 3000 ng cDNAs of *hTRPV3* (gene accession number BC104866.1), *hTRPV1* (accession number NM_080704.3), *mTRPV2* (accession number NM_011706.2), *hTRPV4* (accession number NM_021625.5), *hTRPA1* (accession number NM_007332.3) and *hTRPM8* (accession number NM_024080.5) into HEK293 cells grown in a 35-mm cell culture dish using Lipofectamine 2000 (Invitrogen). Cells were used 18 ∼ 24 h after transfection. All WT or mutant hTRPV3 plasmids were validated by DNA sequencing.

### Electrophysiological recordings

Whole-cell patch clamp recordings of wild-type and mutants were carried out at room temperature using the HEKA EPC10 amplifier powered by PatchMaster software (HEKA) or MultiClamp 700B amplifier driven by Clampex 11.0.3 software. The borosilicate glass pipettes were pulled and fire-polished to 3∼6 MΩ using a DMZ universal electrode puller (Zeitz-Instruments, GmbH). Pipette solution and bath solution both contained (in mM): 130 NaCl, three HEPES, and 0.2 EDTA (pH = 7.4). Cell membrane potential was held at 0 mV, currents were recorded at a voltage ramp from −100 to +100 mV for 500-ms and analyzed at ±80 mV.

For single-channel recordings, borosilicate glass pipettes were pulled from borosilicate glass capillaries and fire-polished to the resistance between 6 and 10 MΩ using a DMZ universal electrode puller (Zeitz-Instruments, GmbH). For inside-out single-channel recordings, both pipette and external solutions contained (in mM): 130 NaCl, three HEPES, and 0.2 EDTA (pH = 7.4). Membrane potential was clamped at −60 mV and currents were digitized at 10 kHz and filtered at 2 kHz.

### Molecular docking and amino acid sequence alignment

Schrödinger Glide (Maestro software suite 2019, Schrödinger) was used to dock flopropione to mTRPV3 (PDB ID code: 6DVY) and mTRPV2 (PDB ID code: 7XEV). The structure of flopropione was obtained from the library of marketed compounds (TargetMol). The compound and docking models were semi-flexibly docked using the built-in program Ligprep after optimization using energy minimization. The binding pose with a docking score greater than −4 was thought to be a possible binding pose of flopropione for TRPV3. Amino acid sequence alignment between mTRPV3 and mTRPV2 and was made using ClustalX2.

### Mouse models of atopic dermatitis and ear swelling induced by chemical DNFB

Mice were placed in a gas anesthesia device (SurgiVet) for anesthesia before the hair on the back was removed with a razor, followed by the application of an appropriate amount of hair removal cream to gently shave off the remaining hair. For generation of atopic dermatitis and ear swelling, chemical skin sensitizer DNFB (1-Fluoro-2,4-dinitrobenzene), commonly known as Sanger’s Reagent, was dissolved in a solvent (acetone/olive oil = 4/1, v/v) and 0.5% DNFB in 100 μl was applied once topically to the back and right ear for skin sensitization 1 day after acclimatization for hair removal (43) without any treatment for next 3 days. On the fifth and sixth days, 0.2% DNFB in 50 μl was applied topically once a day for 2 days for challenge before different concentrations of flopropione in 100 μl were topically applied once a day for four consecutive days. First application of topical flopropione started half an hour after the last application of 0.2% DNFB challenge. For the effect of flopropione on skin inflammation, topical flopropione (0.1 mM, 1 mM or 10 mM) in 100 μl was applied into ear and shaved area of dorsal skin by swabbing once a day for four consecutive days.

For the preventive effect of osthole on the development of ear swelling induced by DNFB, topical osthole (10 mM) was administered 1 day before 0.5% DNFB sensitization until the end for a period of 10 days.

### Dermatitis scoring and ear thickness measurements

The score contents and criteria include (1): skin erythematous/bleeding (2), skin scratches/erosion (3), skin edema (4), skin scaling or lichenoid change (44). Each symptom was scored from 0 to 3 (none, 0; mild, one; moderate, two; severe, (3). The final score was defined as the sum of individual scores on a scale from 0 to 12. Mouse ear thickness was measured once a day for continuous 5 days with the first measurement of ear thickness 30 min after the last application of 0.2% DNFB using vernier caliper.

### Histological sections of skin tissues

Mice were anesthetized with 1.0% isoflurane before sacrifice 24 h after the last treatment (31, 32, 43). Mouse dorsal skin was removed with scissors and forceps before fixed with 4% paraformaldehyde. Paraffin-embedded tissues were sectioned and stained with hematoxylin and eosin. Stained sections were observed using bright-field microscopy (ECLIPSE Ti-S, Nikon) and CCD-camera (DS-Ri2, Nikon).

### Statistical analysis

All data are expressed as the mean ± SD (standard deviation). Paired *t* test and one or two-way ANOVA followed by multiple-comparison test were used to evaluate statistical significance using GraphPad Prism 7.0 and Origin nine software. A value of *p* < 0.05 was considered to be statistically significant.

## Data availability

All data are contained within this paper.

## Supporting information

The article contains supporting information (18).

## Conflict of interest

The authors declare no conflicts of interest in this study.

## Figures and Tables

**Figure S3:**