# Supplementary material for: Selective inhibition of overactive warmth-sensitive Ca2+-permeable TRPV3 channels by antispasmodic agent flopropione for alleviation of skin inflammation
Source: J Biol Chem. 2023 Dec 26;300(2):105595. doi: 10.1016/j.jbc.2023.105595 (PMC10828444; doi:10.1016/j.jbc.2023.105595)
Supplement: Supplementary file 1 — Supplemental figures [file mmc1.docx]

**Supplemental figure legends**

**Figure S1. Comparison of flopropione binding pockets and their amino acids between TRPV3 and TRPV2 channels.**

(A) An overview of putative pocket for flopropione binding to TRPV2 structure (PDB ID code: 7XEV) in a single subunit (Left), with an extended view of flopropione (red) with TRPV2 channel in the pocket formed by three residues Y442, L443 and E665 with a hydrogen bond shown in blue dotted line. (B) Superposition of single subunit in TRPV3-flopropione (yellow) and TRPV2-flopropione (blue) with their extended view. (C) Comparison for alignment of amino acid sequences near the flopropione binding pockets of TRPV3 and TRPV2 proteins. Pentacle indicating identical residues; triangle indicating high similarity; and the blank space indicates a difference between TRPV2 and TRPV3. The residue I497 in cyan box of TRPV3 corresponds to the residue L443 in TRPV2. The residue E501 in red box of TRPV3 corresponds to the Q447 in TRPV2. The height of the gray column indicates the degree of conservatism between TRPV2 and TRPV3.

**Figure S2. Three putative binding pockets for flopropione in TRPV3.**

(A) Flopropione in the binding pocket 1 with putative interaction residues from S1 and S2 of TRPV3 (PDB code: 6DVY).

(B) Flopropione in the binding pocket 2 with putative interaction residues from S6.

(C) Flopropione in the binding pocket 3 with putative interaction residues from S2-S3 linker. Hydrogen bonds are shown in blue dashed lines.

**Figure S3. A putative dyclonine binding pocket primarily formed by residues T665 and F666 from S6 in TRPV3.**

An overview of putative pockets for dyclonine binding to TRPV3 (PDB ID code: 6DVY) from docking in side view (A) and the top-down view (B). The four subunits of the tetramer are distinguished in different colors, with the dyclonine shown in red. (C) An extended view of dyclonine (red) with TRPV3 channel in the pocket formed by T665 and F666 residues from S6 helix. Hydrogen bonds are shown as blue dotted line.
